# Assessing the Effects of Cold Atmospheric Plasma on the Natural Microbiota and Quality of Pork during Storage

**DOI:** 10.3390/foods13071015

**Published:** 2024-03-26

**Authors:** Yelyzaveta K. Oliinychenko, Sotirios I. Ekonomou, Brijesh K. Tiwari, Alexandros Ch. Stratakos

**Affiliations:** 1 School of Applied Sciences, College for Health, Science and Society, University of the West of England, Coldharbour Ln, Bristol BS16 1QY, UK; yelyzaveta2.oliinychenko@live.uwe.ac.uk (Y.K.O.); sotirios.oikonomou@uwe.ac.uk (S.I.E.); 2 Department of Food Biosciences, Teagasc Food Research Centre, Teagasc, Ashtown, D15 DY05 Dublin, Ireland; brijesh.tiwari@teagasc.ie

**Keywords:** non-thermal technologies, food spoilage, antimicrobial effect, shelf life, meat quality

## Abstract

Cold atmospheric plasma (CAP) is a novel non-thermal technology with significant potential for use in meat processing to prolong shelf life. The objective of the study was to evaluate the efficiency of CAP treatment on the natural microbiota and quality traits of pork stored for 8 days at 4 °C. CAP treatment was applied by employing piezoelectric direct discharge technology to treat pork samples for 0, 3, 6, and 9 min. Reductions of approximately 0.8–1.7 log CFU/g were observed in total viable counts (TVC) and *Pseudomonas* spp. levels for CAP treatments longer than 3 min, immediately after treatment. A storage study revealed that CAP-treated pork (>6 min) had significantly lower levels of TVC, *Pseudomonas* spp., and *Enterobacteriaceae* throughout storage. Regarding quality traits, CAP application for longer than 3 min significantly increased water retention and yellowness and decreased meat redness compared to untreated pork. However, other parameters such as pH, tenderness, and lightness exhibited no statistically significant differences between untreated and CAP-treated pork. Lipid oxidation levels were higher only for the 9-min treatment compared to untreated pork. Our results revealed that CAP is a promising technology that can extend the microbiological shelf life of pork during refrigeration storage.

## 1. Introduction

Meat production is currently undergoing major challenges due to increasing global consumption, negatively impacting meat safety and quality, economic profit, and environmental sustainability [1]. Pork is the most consumed red meat worldwide, and its consumption has shown a consistent upward trend in the past few years [2]. Introducing novel processing methods in meat production has been identified as a viable strategy to extend its shelf life and thus reduce food waste [3].

The development of spoilage in pork primarily starts on its surface and is influenced by a range of factors, such as the initial bacterial levels, storage conditions, and processing methods [4]. Pork processors employ a variety of sanitising treatments to decrease microbial populations, including thermal processing and disinfectant application, in accordance with regulations in certain countries [5]. Currently, the methods employed in the processing of pork have shown limited effectiveness and can result in negative effects on the quality of the product (e.g., nutrient loss, cooking loss, shrinkage, colour deterioration, and texture defects) [6]. Thus, there is a critical need to introduce new technologies into the processing of pork that will improve its effectiveness without compromising its quality.

Cold atmospheric plasma (CAP) is currently being investigated as a technology for surface decontamination in meat processing [7]. It is recognised for its antibacterial properties, economic feasibility, and eco-friendliness [8]. Various methodologies, such as dielectric barrier discharge (DBD), plasma jet, corona barrier discharge (CDB), gliding arc discharge, and piezoelectric direct discharge (PDD), can be utilised to generate CAP [9,10]. In the present study, a PDD generator was utilised to produce a cold, non-equilibrium plasma utilising atmospheric air. CAP consists of significant quantities of reactive oxygen species (ROS) and reactive nitrogen species (RNS), which result in the direct damage of bacterial cells [11]. The targeted microorganisms are impacted by the electric field, ROS (hydroxyl radicals, superoxide radicals, singlet oxygen, and hydrogen peroxide), and RNS (nitric oxide, nitrogen dioxide, nitric oxide dimer, and peroxynitrite) by disrupting cellular homeostasis and causing ion imbalance, ultimately resulting in cell death [12].

Considering CAP application to pork when using DBD technology with either helium, argon, or nitrogen gas supply, a prior study reported that the application of CAP in vacuum-packed (VP) pork led to a 0.4 log CFU/g decrease in the total viable counts (TVC) [13]. However, the treatment significantly increased the oxidation of lipids and proteins and reduced redness [13]. Contrary to the mentioned findings [13], another study [14] showed no significant impact of CAP treatment on colour parameters or pH in pork. Also, it was observed that CAP treatment significantly reduced the numbers of TVC (2.0 log CFU/g) and psychrotrophic bacteria (3.0 log CFU/g) in pork [14]. The studies focusing on the antibacterial efficiency of CAP against pathogens in pork (e.g., *Escherichia coli*, *Listeria monocytogenes*, and *Salmonella* Typhimurium) led to a reduction up to 3.0 log CFU/g [15,16,17]. While CAP is efficient against spoilage and pathogenic bacteria, it is not employed in meat processing. According to Vukić et al. [18], only a small proportion of academic studies conducted in 2021 (7%) and 2022 (8%) have focused on applying CAP in meat processing when compared to other cold plasma applications. This shows that there is limited knowledge, and more research is necessary to investigate the effect of CAP on the shelf life and quality of meat products. Consequently, the impact of CAP treatment on the natural microbiota (initially present in pork) and quality of pork has not been fully investigated.

In the current study, we aimed to determine the effect of CAP producedusing PDD technology on pork natural microbiota levels during refrigeration storage (4 °C) and its impact on meat quality traits. The application of PDD technology to produce CAP has not yet been investigated in pork meat processing. Specifically, we assessed the effect of CAP treatment on TVC, LAB, *Enterobacteriaceae*, and *Pseudomonas* spp. at different treatment durations and evaluated the impact of CAP on pH, tenderness, water-holding capacity, cooking loss, colour parameters, and thiobarbituric reactive substances (TBARS) levels.

## 2. Materials and Methods

### 2.1. Experimental Set-Up

Pork loins (*M. longissimus thoracis et lumborum*) were purchased from a single retail supplier (Sainsbury’s, a UK supermarket chain). Three different pork loin batches were purchased from the same supplier with the same expiration date and immediately stored at 4 °C. An initial assessment (as detailed in Section 2.3) was performed to exclude loins of poor quality. Subsequently, the loins were meticulously cut into 10 g square-shaped pieces under aseptic conditions. All experiments were performed in triplicate with two technical replicates.

### 2.2. Cold Atmospheric Plasma (CAP) Treatment

The application of CAP was conducted using a piezoelectric direct discharge CAP generator (PiezoBrush PZ3, Relyon Plasma, Regensburg, Germany) with an input voltage of 12 V, a frequency of 50 kHz, and atmospheric air as the gas source, propelled by a fan in order to transfer the excited species and chemical radicals toward the matrix surface. The generator operated 10 mm from the sample surface. Each sample was carefully placed in a labelled, sterile petri dish before undergoing CAP treatment. Various treatment durations (0, 1, 3, 6, and 9 min) were applied. Both treated and untreated samples were VP in 15 × 15 cm sealing bags utilising a Deep Chamber Vacuum Sealer (SousVideTools, Lancaster, UK). The VP samples were then stored at 4 °C and subjected to analysis at predefined time intervals (0, 2, 4, 6, and 8 days) during refrigeration storage. All experiments were conducted in triplicate with two technical replicates.

### 2.3. Meat Sample Standardisation

Due to the substantial influence of pork quality on spoilage development [19], only pork loin steaks meeting the defined normal quality criteria were included in the experiment. The criteria for normal pork quality were based on a pH range of 5.4 to 5.9 (measured 48 h after packing) and an L* colour parameter range of 49 to 59, as established by Faucitano et al. [19]. In line with these studies, pork loin samples exhibiting a pH within this range and an L* colour parameter within the specified spectrum were considered to possess normal quality.

### 2.4. Microbiological Analysis

Meat samples were aseptically unpacked, cut into 10 g pieces, and transferred into sterile stomacher bags with 90 mL maximum recovery diluent (MRD) and blended with a stomacher (400 Circulator Lab Blender, Seward Ltd., Cambridgeshire, UK) for 2 min at 240 rpm [20]. The samples were prepared in a series of decimal dilutions with sterile MRD, and each dilution was spread or pour-plated into a Petri dish with the appropriate growth medium depending on the studied microorganism. Total microbial counts (TVC) were enumerated using Plate Count Agar (PCA, Oxoid, CM0463, Basingstoke, UK) at 32 °C for 48 h incubation. Lactic acid bacteria (LAB) counts were measured using De Man–Rogosa–Sharpe agar (MRS, Oxoid, CM1153) at 30 °C for 48 h incubation, and *Enterobacteriaceae* counts were enumerated with Violet Red Bile Glucose Agar (VRBGA, Oxoid, CM1082) at 37 °C for 24 h incubation. Moreover, *Pseudomonas* spp. were determined using Pseudomonas Agar Base (Oxoid, CM0559) with the added cephaloridine fucidin cetrimide-selective agar supplement (CFC, Oxoid, CM0559) at 25 °C for 48 h incubation. After incubation, the colonies were counted, and the bacterial populations were calculated based on the appropriate dilution factors. Microbial counts were determined by quantifying log colony-forming units (CFU) per gram (CFU/g) in pork samples following Equations (1) and (2).

(1) $$CFU/g=\frac{Number\;of\;colonies\;counted}{Dilution\;factor\times Volume\;plated\;(in\;mL)}$$

(2) Log CFU/g=log10(CFU/g)/(Grams of sample)

### 2.5. Determination of Meat Quality

#### 2.5.1. Analysis of Tenderness

Tenderness was analysed using a TA.XTplus Texture Analyser (Stable Micro Systems, Surrey, UK) equipped with a load cell of 5 kg with a Warner–Bratzler standard blade (Stable Micro Systems), following Choe et al.’s [21] protocol. Briefly, a 2.0 × 2.0 × 2.0 cm meat sample was positioned beneath the blade on the stand. The analysis involved a pre-test speed of 3.0 mm/s, followed by a test speed of 1.0 mm/s, and finally a post-test speed of 3.0 mm/s. The maximum shear force required to cut the sample was measured to quantify its tenderness (in kg).

#### 2.5.2. Analysis of pH

The pH of meat was measured after homogenising 10 g of meat with 90 mL of distilled water using a pH metre (HI-5221 pH metre, Kisker, Steinfurt, Germany) [16]. Prior to conducting measurements, the pH metre was calibrated using standard buffer solutions (pH 4.00, 7.00, and 10.00).

#### 2.5.3. Analysis of TBARS

Secondary lipid oxidation products were quantified following the protocols of Ekonomou et al. [20] and Pomponio and Ruiz-Carrascal [22], with some modifications. In summary, 5 g of the sample was homogenised in 15 mL of 7.5% trichloroacetic acid (TCA) containing 0.10% propylgallate and 0.10% ethylenediaminetetraacetic acid (EDTA) using a homogeniser (SHM1 Handheld Homogeniser, Stuart, Staffordshire, UK) at 13,500 rpm for 1 min. The homogenate was filtered and combined with 2.5 mL of 20 mM thiobarbituric acid (TBA). The mixture was then incubated in a water bath at 100 °C for 40 min.

Absorbance readings were taken at 532–600 nm. TBARS results represent the means of two replicates from the same sample (30 samples on each day of storage) and are expressed in milligrams of malondialdehyde (MDA) per kilogram of the sample. The quantification was performed using a standard curve generated from a known concentration of MDA standard.

#### 2.5.4. Cooking Loss

Cooking loss (%) was determined according to Abdel-Naeem et al.’s [23] protocol by evaluating the sample weight loss following Equation (3), where Wr represents the raw weight of the sample and Wc represents the cooked weight of the sample.

(3) $$Cooking\;loss\;(\%)=\frac{\left(Wr-Wc\right)}{Wc\times 100}$$

#### 2.5.5. Drip Loss

Drip loss was measured according to the Jankowiak, Cebulska, and Bocian [24] protocol. A sample (300 mg) was placed under Whatman filter paper 1 between two glass plates under a constant load of 2 kg for 5 min. The expressed juice area was used to calculate the percentage of loose water in the sample when 1 cm^2^ of infiltration corresponds to 10 mg of water. The expressed juice area was measured with the ImageJ software program (Version 1.8.0).

#### 2.5.6. Analysis of Colour

The colour parameters L*, a*, and b* were evaluated according to Choi, Puligundla, and Mok’s [15] protocol using the Konica Minolta CR 410 spectrophotometer (Marunouchi, Osaka, Japan). Briefly, the device was put against the pork sample in four different spots on each side of the sample. The mean value of four readings was calculated, and the device underwent calibration using a white calibration plate prior to each experimental batch.

### 2.6. Statistical Analysis

The experiments evaluating the effects of CAP treatments (0, 1, 3, 6, and 9 min) on pork spoilage and quality during 8-day storage were performed in triplicate, with each set repeated two times. Statistical analysis was conducted using Minitab Statistical Software (https://www.minitab.com/en-us/products/minitab/), Minitab Inc., employing Tukey’s post hoc analysis at a significance level of *p* < 0.05. Mean comparisons were carried out using two-way ANOVA analysis (with CAP treatment and storage time as independent variables), and the data were presented as mean values ± standard deviation.

## 3. Results and Discussions

### 3.1. Impact of CAP Treatment on Spoilage Microbiota in Pork during Storage

**Figure 1:**
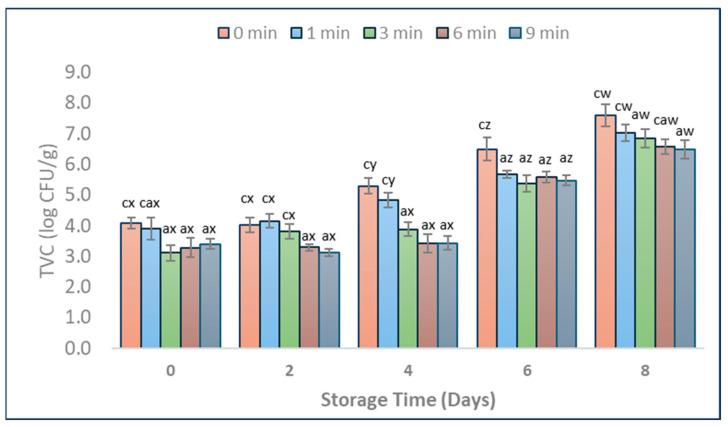
TVC in pork samples (vacuum packed, 0–8 days of storage at 4 °C) with different durations of CAP treatment (1, 3, 6, and 9 min) and untreated ones (0 min). Populations are shown as log transformations to base 10. Letters a and c indicate means that differ significantly (*p* < 0.05) between CAP treatment times on the same day. Letters w, x, y, and z indicate significant mean differences (*p* < 0.05) through storage within the same treatment. Error bars indicate the standard deviation; each treatment time was repeated in triplicates, and each set was replicated two times, resulting in n = 6.

**Figure 2:**
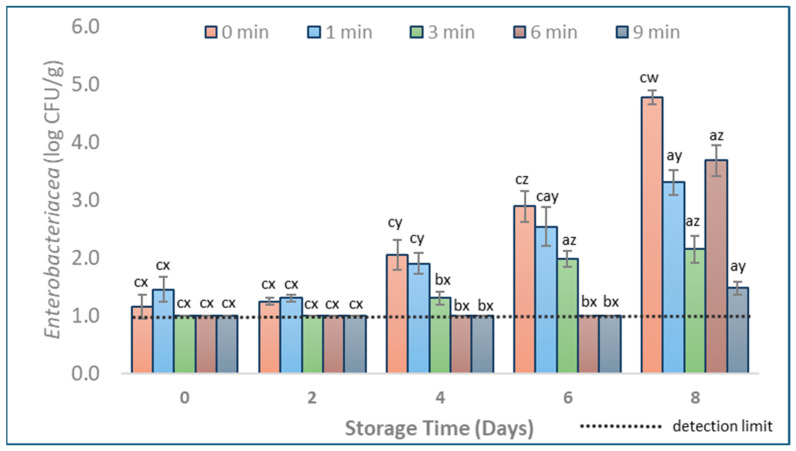
*Enterobacteriaceae* mean populations in pork samples (vacuum packed, 0–8 days of storage at 4 °C) with different durations of CAP treatment (1, 3, 6, and 9 min) and untreated ones (0 min). Populations are shown as log transformations to base 10. Letters a, b, and c indicate means that differ significantly (*p* < 0.05) between CAP treatment times on the same day. Letters w, x, y, and z indicate significant mean differences (*p* < 0.05) through storage within the same treatment. Error bars indicate the standard deviation; each treatment time was repeated in triplicates, and each set was replicated two times, resulting in n = 6. The detection limit was 1.0 log CFU/g.

This study presents an in-depth approach to assessing the effects of CAP treatment on pork, with a particular focus on its impact on the natural microbiota and the overall quality of the meat. TVC and *Enterobacteriaceae* levels in CAP-treated (1, 3, 6, and 9 min) and untreated (0 min) pork samples on different days of storage are represented in Figure 1 and Figure 2. The higher reductions of TVC levels immediately after treatment were achieved for 3, 6, and 9 min CAP treatments when compared to the untreated samples. CAP treatment for 1 min did not significantly affect TVC levels when compared to untreated samples (Figure 1, *p* > 0.05). *Enterobacteriaceae* levels were reduced below the detection limit (1.0 log CFU/g) when CAP was applied for at least 3 min. Samples treated for 6 and 9 min showed significantly lower (Figure 2, *p* < 0.05) TVC and *Enterobacteriaceae* levels throughout storage compared to untreated samples. The 3 min CAP treatment led to a significant reduction of TVC levels (up to 1.4 log CFU/g reduction) on Days 0, 4, and 6, as well as *Enterobacteriaceae* levels (up to 2.7 log CFU/g reduction) on Days 4, 6, and 8 (Figure 1 and Figure 2).

Ulbin-Figlewicz, Brynchcy, and Jarmoluk [14] found a significant decrease of 1.3 log CFU/g in TVC counts in pork meat after 5 min of CAP treatment when utilising DBD with helium and argon as gases for igniting the plasma. When the exposure time was extended to 10 min, it was observed that TVC decreased by approximately 2.0 log CFU/g. Ulbin-Figlewicz, Brynchcy, and Jarmoluk [14] found that the effectiveness of CAP varied depending on the main gas supply; as a result, nitrogen CAP treatment had no significant impact on TVC counts in pork, unlike with helium and argon plasma. The difference in main supply gases leads to varying electronegativities, ionisation potentials, and affinities of different gases, which affect the types and quantities of ions and radicals in CAP and have diverse effects on bacteria [10].

Regarding the reduction of *Enterobacteriaceae*, our results are in agreement with those reported by Sammanee et al. [25] and Moutiq et al. [26] studies, which used plasma-activated water (PAW) and CAP (DBD, in-package treatment) to treat pork and chicken, respectively. However, application of PAW in the Sammanee et al. [25] study did not lead to any significant reduction of *Enterobacteriaceae* in pork samples, in contrast to our results. Such differences are likely related to using different plasma sources (gas versus liquid) and main gas sources, such as atmospheric air versus argon, or even using different meat parts (e.g., loin and pork belly).

**Figure 3:**
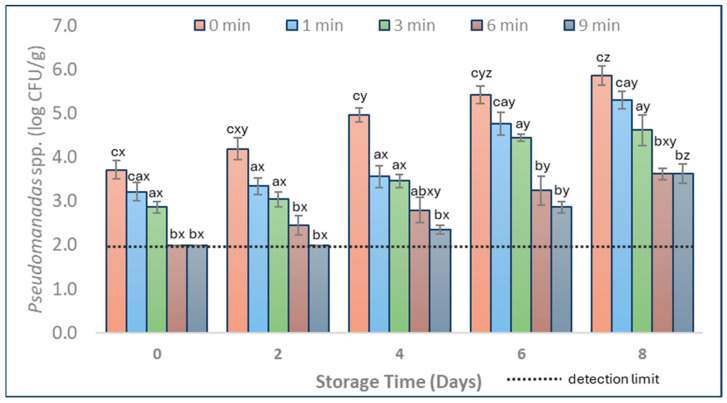
*Pseudomonas* spp. mean populations in pork samples (vacuum packed, 0–8 days of storage at 4 °C) with different durations of CAP treatment (1, 3, 6, and 9 min) and CAP untreated ones (0 min). Populations are shown as log transformations to base 10. Letters a, b, and c indicate means that differ significantly (*p* < 0.05) between treatment times on the same day. Letters x, y, and z indicate significant mean differences (*p* < 0.05) through periods of storage within the same treatment. Error bars indicate the standard deviation; each treatment time was repeated in triplicates, and each set was replicated two times, resulting in n = 6. The detection limit was 2.0 log CFU/g.

**Figure 4:**
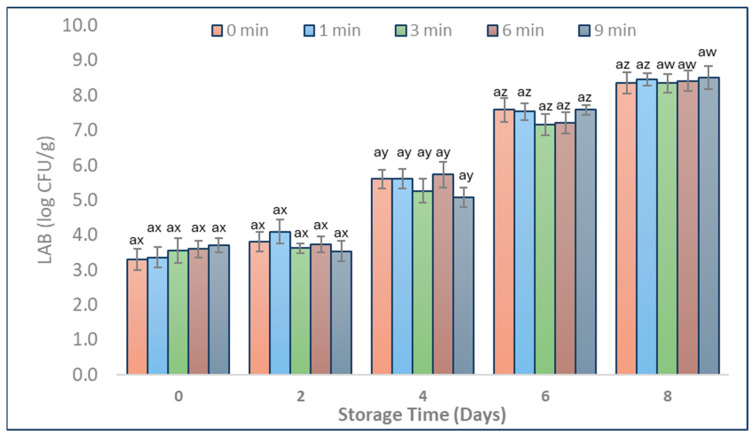
LAB counts in pork samples (vacuum packed, 0–8 days of storage at 4 °C) with different durations of CAP treatment (1, 3, 6, and 9 min) and CAP untreated ones (0 min). Populations are shown as log transformations to base 10. Letter a indicates no significant differences (*p* > 0.05) between treatments on the same day. Letters x, y, z, and w indicate significant mean differences (*p* < 0.05) through storage within the same treatment. Error bars indicate the standard deviation; each treatment time was repeated in triplicates, and each set was replicated two times, resulting in n = 6.

*Pseudomonas* spp. and LAB have been identified by other studies as being among the main representatives of the natural microbial composition of pork during refrigeration storage [3,27]. The mean populations of *Pseudomonas* spp. and LAB counts in CAP-treated (1, 3, 6, and 9 min) and untreated (0 min) pork during storage are shown in Figure 3 and Figure 4.

Except for the 1 min treatment on Day 0, CAP application significantly decreased the population of *Pseudomonas* spp. throughout storage at 4 °C. On Day 0, *Pseudomonas* spp. was reduced below the detection limit (2.0 log CFU/g) after 6 and 9 min CAP treatments. The results showed that CAP treatment for 6 and 9 min was more effective in reducing *Pseudomonas* spp. in pork than for 1 and 3 min (Figure 3, *p* < 0.05). On Days 2, 4, 6, and 8, *Pseudomonas* spp. levels were lower by over 2.0 log CFU/g after 6 and 9 min treatments compared to the untreated samples (Figure 3, *p* < 0.05). These findings suggest that a minimum treatment of 6 min is necessary to reduce *Pseudomonas* spp. in pork. Our findings support those of Zhang et al. [28], who found that applying DBD technology (argon plasma) for 5 min reduced *Pseudomonas* spp. levels by 2.0 log CFU/g when tested in chicken.

Our study’s findings suggest that the use of CAP did not significantly impact the LAB counts in pork (Figure 4, *p* > 0.05) when using PDD technology. On Day 2, LAB counts showed no significant increase compared to Day 0 for all tested CAP treatment durations (Figure 4). Zhang et al. [28] reported that CAP treatment using a DBD device for up to 5 min significantly decreased LAB in chickens. In addition, CAP treatment using DBD technology for 3 min significantly reduced LAB counts by 0.6 log CFU/g on fresh fish (*Trachinotus ovatus*) stored at 4 °C under modified atmosphere packaging [7]. A similar decrease in LAB counts was observed in our study on Day 4, where the CAP-treated samples for 3 and 9 min revealed a statistically insignificant reduction of approximately 0.5 log CFU/g compared to untreated samples (Figure 4, *p* > 0.05). To our knowledge, no research has been conducted on the impact of CAP on the composition of LAB in pork. Our results revealed that CAP treatment for up to 9 min did not inhibit LAB growth in pork during storage at 4 °C for 8 days (Figure 4). In contrast to our results, other studies showed reduced LAB counts after utilising various CAP technologies on other food products such as fish, chicken, and different parts of pig carcass versus pork loin samples [7,28]. This can be due to the utilisation of different CAP generators that can lead to different antimicrobial efficiency due to diverse configurations and processing conditions involving different gas supplies (helium, argon, nitrogen, helium, and atmospheric air), varying operating pressures, electrode configurations, power input, and gas flow rates [10].

The clear antimicrobial effect observed on the natural microbiota of pork meat can be attributed to the presence and accumulation of reactive oxygen and nitrogen species present. Based on prior investigations into the composition of PDD plasma, Timmermann et al. [29] and Korzec, Hoppenthaler, and Nettesheim [30] found that the atmospheric air-produced plasma primarily consists of ozone, nitrogen dioxide, and nitric oxide. At a molecular level, the plasma reactive species cause the breaking of chemical bonds within the cellular membrane, particularly those of C-O and C-C bonds, leading to significant structural damage [12,31,32]. On a cellular level, CAP causes damage to the bacterial cell membrane, DNA, and proteins, ultimately leading to cell death. According to Chauvin et al. [33] and Nicol et al. [12], due to the damage inflicted on the cell membrane, the reactive species can also enter the cell, where they interact with essential intracellular components, causing further damage to the cell.

### 3.2. Impact of CAP Treatment on Meat Quality in Pork during Storage

#### 3.2.1. pH

The pH value is a key meat quality parameter that indicates the concentration of hydrogen ions, which constantly changes due to ongoing anaerobic metabolic processes [34]. Moreover, pH value is the main trait that defines pork quality, as it affects the ability of meat to retain water, colour, and tenderness [24]. The pH of CAP-treated and untreated pork during storage is summarised in Table 1. On Day 0, the pH (48 h after packaging) for all experimental groups was within the range of 5.38–5.41, corresponding to pork of normal quality [27]. Considering the storage impact, there was a statistically significant pH drop in all experimental groups on Days 6 and 8 compared to Day 0 for the untreated samples (Table 1, *p* < 0.05). At this point, the population of microorganisms increased, leading to higher rates of glycogen breakdown that can cause pH reduction [27]. At the end of the storage period (Day 8), there were no significant differences in the pH value between the treated (9 min treatment) and untreated pork samples. Our results are consistent with those of Jung et al. [35], who found no significant effect of CAP on the pH of processed pork after utilising DBD for 5–20 min. Overall, the absence of an effect of CAP treatment on pH value can be considered beneficial because meat’s pH value significantly impacts overall meat quality. More research is required to fully comprehend the mechanism causing the pH changes in fresh pork meat after CAP treatment.

#### 3.2.2. Cooking Loss

Cooking loss indicates the ability of meat to retain its water after heating and is an important parameter as it defines meat juiciness and overall quality [24]. Furthermore, the meat industry suffers economic losses due to excessive weight loss caused by increased cooking losses [36]. Following the application of CAP, a notable reduction in cooking loss was observed in the treated pork compared to the untreated ones, as indicated in Table 1. Furthermore, the prolonged CAP treatment time resulted in a proportional decrease in cooking loss. The application of CAP for 6 and 9 min yielded a notable reduction in cooking loss compared to untreated pork during storage, which is a favourable outcome for enhancing product quality and reducing potential economic losses in the processing chain. This result highlights the potential of CAP treatment as an effective intervention for pork processing.

#### 3.2.3. Water-Holding Capacity

Water-holding capacity (WHC) indicates the ability of meat to hold water during storage and after the application of any external forces (e.g., heating, cutting) [24]. WHC was found to be significantly higher (*p* < 0.05) in 6 and 9 min CAP-treated pork on Days 2, 4, and 6 (Table 1) when compared to untreated samples. WHC in 3 min CAP-treated pork was found to increase only on Day 4, which shows that 6 and 9 min CAP treatments have a major impact on WHC. According to Luo et al. [37], the higher WHC in CAP-treated pork could be explained by myofibril structure damage. For instance, after CAP treatment, Luo et al. [37] observed more fractured Z-lines and fewer distinct M-lines in pork. Consequently, alterations to the myofibril structure (after CAP application) could alter the isoelectric point in myofibrils, leading to an increase in meat water retention and a higher WHC [37].

#### 3.2.4. Water Retention in Meat during Storage

Water retention refers to the ability of meat to retain water during storage, processing, and cooking [38]. Water retention plays a major role in reducing the weight of meat during cooking or storage [39], affecting the general quality of meat as well as the economic sustainability of production. To assess the water retention in pork, we focused on the examination of cooking loss and WHC [40]. Throughout the 8-day storage, there was a significant increase in cooking loss of samples of all experimental groups compared to Day 0 (Table 1) and throughout storage (Days 2, 4, 6, and 8). WHC (Table 1) showed a comparable trend, when all samples on Day 0 revealed significantly lower WHC than samples from Days 2 to 8. These findings align with the results of Park et al. [41], who observed a similar reduction in WHC in VP-pork during refrigeration storage. The ongoing anaerobic processes happening in VP-meat during storage, such as anaerobic glycolysis and bacterial decomposition, could decrease pork’s overall water retention [42]. Anaerobic glycolysis and bacterial decomposition can also lead to the accumulation of a significant quantity of lactic acid and nitrogenous waste, causing a significant decrease in meat pH [24]. Consequently, a decrease in pH affects the overall charge of the main proteins found in muscle, leading to changes in the chemical and physical characteristics of proteins. After a drop in pH, the muscles contract and gradually lose their ability to retain water, which could explain the results observed in our study [42]. Our results suggest that CAP treatment did not affect the pH of pork meat, and any changes caused during refrigeration storage were due to bacterial growth. This highlights that CAP can be used without affecting the WHC of pork meat.

#### 3.2.5. Tenderness

Tenderness is one of the most important meat quality traits influencing consumer acceptability [34]. Lack of meat tenderness is frequently stated as the primary reason for consumer dissatisfaction [43].

The data from Table 1 indicate that CAP treatment did not have a statistically significant impact on the tenderness of pork (*p* > 0.05) during storage. Our results align with those of Xiang et al. [9], who found that a 10 min DBD treatment did not have any significant effect on the tenderness of pork. In contrast, Luo et al. [37] observed that the application of CAP (DBD) for 5 min at a voltage of 70 kV improved the tenderness of pork when compared to untreated pork and CAP-treated pork with lower voltages (50 and 60 kV). The observed trend in the Luo et al. [37] study can be attributed to a notable reduction in the pH value of pork treated with a 5 min CAP treatment at 70 kV voltage. The mentioned pH reduction might have reduced myofibers’ ability to retain water, which could lead to a significant increase in tenderness. However, our study found no evidence of CAP treatment impacting the pH level of pork during storage (Section 3.2.1), which could potentially account for the lack of changes in tenderness.

#### 3.2.6. Colour Parameters

The colour parameters of all experimental groups are shown in Table 2. The L* value (lightness) was not affected by the CAP application on any day of storage. Contrary to our findings, Jayasena et al. [17] found that the L* value for plasma-treated pork (i.e., utilising DBD and nitrogen/oxygen plasma for 5 min) significantly decreased when compared to untreated samples. The a* value (redness) significantly decreased only in pork treated with CAP for 6 and 9 min on Days 0 and 2 (Table 2). Huang et al. [13] reported similar results for the a* value being decreased for CAP-treated pork yellowness (b*) of CAP-treated samples significantly increased on Day 0 when compared to the untreated group. However, on Day 9, the b* value significantly increased only at 9 min for CAP-treated pork when compared to untreated samples.

Considering the impact of CAP treatment time, the b* value of pork treated for 1 min was significantly lower than the b* value of 9 min CAP-treated pork. The current study’s findings align with those of Luo et al. [37], who also observed an increase in the b* value when pork was treated with CAP using DBD technology for a duration of 3 min. In other studies, however, it was reported that the b* value of CAP-treated pork decreased [13]. Different plasma flow and physicochemical characteristics may account for variations in colour parameters observed in previous studies employing various CAP generation technologies (DPP, DBD, and DBD-CP) and processing conditions (time, application distance, etc.) [10].

Considering consumers’ perceptions, Ngapo et al. [44] found that the redness of pork is the primary factor influencing the perception of colour; very pale meat is perceived to be of low quality. The L* value, rather than the a* value, is used to determine the redness of pork. When classifying pork into quality classes (such as red, firm, and non-exudative (RFN); pale, soft, and exudative (PSE); and dark, firm, and dry (DFD)), for instance, only the range for the L* value is determined [19,37]. Therefore, the non-significant change in the L* values of CAP-treated pork observed here is considered a positive result in terms of consumer perception [44]. Overall, the mechanism by which CAP affects the colour of pork meat can vary depending on factors such as treatment parameters, meat composition, and environmental conditions. Further investigation is required to understand the chemical reactions and oxidative processes involved during CAP treatment to help overcome this issue.

#### 3.2.7. Lipid Oxidation

TBARS levels are commonly used for the quality and freshness evaluation of meat as they indicate lipid oxidation levels [45]. Lipid oxidation is a natural process that occurs in meat products and refers to the oxidative degradation of fats or lipids present in the meat. This process can lead to changes in the colour, texture, and flavour of the meat, which can ultimately affect its quality and determine the end of its shelf life [46]. The oxidation of lipids in meat can be caused by a variety of factors, including exposure to oxygen, heat, light, enzymes, and CAP treatment [13].

**Figure 5:**
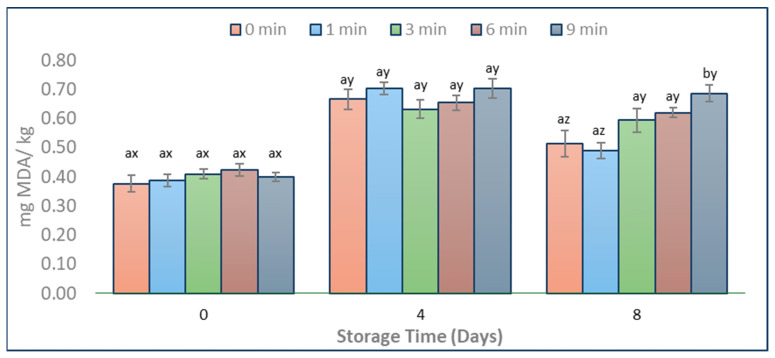
TBARS levels in CAP-treated (1 min, 3 min, 6 min, and 9 min) and untreated (0 min) pork stored at 4 °C. The unit of measure for TBARS is malondialdehyde (MDA) mg/kg. Letters a and b indicate means that differ significantly (*p* < 0.05) between CAP treatments within the same day. Letters x, y, and z indicate means that differ significantly (*p* < 0.05) through the different days of storage within the same treatment. Error bars indicate the standard deviation; each treatment time was repeated in triplicates, and each set was replicated two times, resulting in n = 6.

During storage, TBARS levels significantly increased in all experimental groups, and the values ranged from 0.4 to 0.8 mg MDA/kg, which agrees with the results of Kim et al. [16] for VP-pork. Immediately after treatment (Day 0), there was no significant difference in TBARS values between CAP-treated and untreated pork (Figure 5). However, there was a significant increase in TBARS levels for the 9 min CAP-treated pork at the end of storage (Day 8). This finding is consistent with the research conducted by Kim et al. [16], who found increased TBARS values in VP-pork treated with CAP (DBD, >10 min) when compared to untreated ones at the end of refrigeration storage.

Our study revealed that only a 9 min CAP treatment led to a significant increase in TBARS levels in pork. A potential cause could be that the exposure time (1, 3, and 6 min) was insufficient to initiate oxidative stress, in contrast to the 9 min CAP treatment. Furthermore, the non-significant impact on lipid oxidation of plasma treatments on Day 0 and Day 4 can be attributed to the limited permeability of plasma [40] and the relatively low intramuscular fat content in pork loin [47] compared to other parts of the carcass.

## 4. Conclusions

The study examined the efficacy of CAP treatment in reducing levels of potential spoilage microorganisms in vacuum-packaged pork stored at 4 °C. CAP significantly decreased TVC, *Enterobacteriaceae*, and *Pseudomonas* spp. levels, extending the pork’s microbiological shelf life. Although no significant changes were observed in pH, tenderness, or L* colour, changes in a* and b* colour parameters, along with increased lipid oxidation levels for the longer CAP treatment at the end of the storage period, were noted. These findings show CAP’s potential for extending pork’s microbiological shelf life in a sustainable manner. Future research should explore the effectiveness of utilising natural antioxidant agents or novel packaging techniques (e.g., active packaging) to counteract lipid oxidation. Moreover, future research could focus on further optimising the CAP processing conditions to mitigate these minor quality effects and evaluate their effects on the organoleptic properties of pork.

## Figures and Tables

**Table 1:** Meat quality parameters for pork treated with CAP for varying exposure times (1, 3, 6, and 9 min) and untreated with CAP (0 min).

	Trait	CAP Treatment Time				
		0 min	1 min	3 min	6 min	9 min
Day 0	pH	5.41 ± 0.02 ^azy^	5.40 ± 0.05 ^azy^	5.40 ± 0.04 ^az^	5.38 ± 0.02 ^azy^	5.39 ± 0.01 ^azy^
	cooking loss, %	18.51 ± 0.52 ^aw^	17.32 ± 0.53 ^acw^	15.59 ± 0.55 ^cbw^	15.61 ± 0.48 ^cbw^	15.61 ± 0.65 ^bv^
	WHC, %	16.00 ± 0.82 ^az^	16.45 ± 0.83 ^az^	16.31 ± 0.73 ^az^	16.19 ± 0.81 ^az^	16.91 ± 0.71 ^az^
	Tenderness, kg	2081.10 ± 388.26 ^az^	1848.17 ± 364.31 ^az^	1760.44 ± 318.32 ^az^	1988.35 ± 261.73 ^aw^	2069.22 ± 376.28 ^az^
Day 2	pH	5.42 ± 0.03 ^az^	5.41 ± 0.03 ^az^	5.41 ± 0.04 ^az^	5.42 ± 0.04 ^az^	5.40 ± 0.03 ^az^
	cooking loss, %	19.31 ± 0.47 ^az^	19.34 ± 0.30 ^az^	18.73 ± 0.43 ^ayz^	18.29 ± 0.42 ^bz^	14.53 ± 0.22 ^bw^
	WHC, %	15.61 ± 0.46 ^ay^	15.90 ± 0.63 ^ay^	16.42 ± 0.48 ^by^	16.49 ± 0.49 ^by^	16.48 ± 0.61 ^by^
	Tenderness, kg	2199.51 ± 223.11 ^ay^	2605.47 ± 226.90 ^ay^	2219.48 ± 194.66 ^ay^	2637.76 ± 268.33 ^az^	2872.79 ± 295.60 ^ay^
Day 4	pH	5.37 ± 0.04 ^ayx^	5.36 ± 0.05 ^ayx^	5.35 ± 0.02 ^ay^	5.37 ± 0.05 ^ayx^	5.37 ± 0.03 ^ayx^
	cooking loss, %	19.33 ± 0.31 ^az^	18.17 ± 0.25 ^abx^	17.50 ± 0.41 ^abx^	16.17 ± 0.49 ^bdx^	13.08 ± 0.40 ^dx^
	WHC, %	15.37 ± 0.62 ^ax^	15.10 ± 0.35 ^ax^	16.19 ± 0.71 ^ax^	16.89 ± 0.71 ^bx^	17.12 ± 0.74 ^bx^
	Tenderness, kg	2415.03 ± 323.65 ^ax^	2212.67 ± 451.21 ^ax^	2281.69 ± 127.03 ^ax^	2398.31 ± 502.11 ^ay^	2846.33 ± 611.57 ^ax^
Day 6	pH	5.32 ± 0.04 ^axw^	5.33 ± 0.04 ^ax^	5.37 ± 0.02 ^azy^	5.33 ± 0.03 ^ax^	5.35 ± 0.40 ^ax^
	cooking loss, %	19.18 ± 0.54 ^ay^	17.32 ± 0.51 ^aw^	18.15 ± 0.52 ^ay^	15.54 ± 0.36 ^bw^	15.73 ± 0.39 ^by^
	WHC, %	14.21 ± 0.45 ^aw^	14.32 ± 0.65 ^aw^	14.70 ± 0.43 ^aw^	14.89 ± 0.44 ^bw^	15.11 ± 0.54 ^bw^
	Tenderness, kg	2432.51 ± 58.34 ^aw^	2400.23 ± 183.24 ^aw^	2608.23 ± 232.67 ^aw^	2545.17 ± 189.28 ^ax^	2601.76 ± 365.17 ^aw^
Day 8	pH	5.35 ± 0.03 ^aw^	5.36 ± 0.04 ^ayx^	5.34 ± 0.04 ^ay^	5.36 ± 0.04 ^ayx^	5.36 ± 0.03 ^ax^
	cooking loss, %	19.07 ± 0.28 ^ax^	18.34 ± 0.40 ^ay^	18.76 ± 0.31 ^az^	16.68 ± 0.57 ^by^	16.37 ± 0.50 ^bz^
	WHC, %	12.89 ± 0.46 ^av^	13.10 ± 0.61 ^av^	13.45 ± 0.46 ^av^	13.64 ± 0.39 ^av^	13.54 ± 0.71 ^av^
	Tenderness, kg	1963.18 ± 260.52 ^av^	2065.69 ± 166.43 ^av^	1827.01 ± 247.50 ^av^	1976.72 ± 337.98 ^aw^	2049.52 ± 228.13 ^av^

Letters a, b, and c indicate means that differ significantly (*p* < 0.05) between different CAP treatment times within the same day. Letters v, w, x, y, and z indicate means that differ significantly (*p* < 0.05) through the different days of storage within the same treatment. Error bars indicate the standard deviation; each treatment was repeated in triplicates, and each set was replicated two times, resulting in n = 6.

**Table 2:** Colour parameters of pork treated with CAP for varying exposure times (1, 3, 6, and 9 min) compared to untreated pork (0 min).

	Colour Spec		CAP Treatment			
		0 min	1 min	3 min	6 min	9 min
Day 0	L*	50.13 ± 1.73 ^az^	52.98 ± 1.21 ^az^	51.99 ± 2.71 ^az^	51.18 ± 0.97 ^az^	52.77 ± 2.39 ^az^
	a*	4.26 ± 0.23 ^ay^	3.53 ± 0.94 ^ax^	2.74 ± 0.42 ^bz^	2.25 ± 0.41 ^bw^	2.76 ± 0.56 ^bz^
	b*	7.18 ± 0.44 ^az^	8.25 ± 0.49 ^bz^	7.94 ± 0.21 ^bz^	8.15 ± 0.35 ^bz^	8.36 ± 0.42 ^bz^
Day 2	L*	53.10 ± 4.26 ^ay^	56.01 ± 0.82 ^ay^	55.65 ± 2.79 ^ay^	53.80 ± 1.56 ^ay^	54.14 ± 1.58 ^ay^
	a*	3.76 ± 0.40 ^aw^	3.08 ± 0.38 ^bw^	2.82 ± 0.58 ^by^	2.24 ± 0.23 ^bw^	2.33 ± 0.15 ^by^
	b*	4.78 ± 0.31 ^aw^	4.97 ± 0.21 ^aw^	5.01 ± 0.52 ^aw^	5.34 ± 0.31 ^ay^	5.66 ± 0.41 ^bw^
Day 4	L*	57.35 ± 1.97 ^ax^	58.35 ± 2.14 ^ax^	58.87 ± 0.98 ^ax^	58.06 ± 1.17 ^ax^	59.03 ± 3.48 ^ax^
	a*	4.24 ± 0.87 ^ayx^	3.88 ± 0.19 ^ay^	3.78 ± 0.47 ^ax^	3.47 ± 0.64 ^ax^	3.25 ± 0.22 ^bx^
	b*	5.07 ± 0.05 ^ax^	4.97 ± 0.17 ^aw^	4.98 ± 0.43 ^aw^	5.03 ± 0.32 ^ax^	5.71 ± 0.24 ^bw^
Day 6	L*	59.28 ± 2.96 ^aw^	59.34 ± 0.49 ^aw^	60.39 ± 2.82 ^aw^	60.86 ± 1.19 ^aw^	57.51 ± 1.83 ^aw^
	a*	4.72 ± 0.56 ^az^	4.18 ± 0.70 ^az^	4.14 ± 0.12 ^aw^	4.05 ± 0.76 ^az^	4.10 ± 0.65 ^aw^
	b*	5.91 ± 0.50 ^ay^	6.17 ± 0.15 ^ay^	6.48 ± 0.59 ^ay^	6.48 ± 0.53 ^aw^	6.67 ± 0.27 ^by^
Day 8	L*	61.19 ± 2.60 ^av^	60.70 ± 2.76 ^av^	59.86 ± 2.51 ^av^	59.30 ± 2.82 ^av^	59.53 ± 1.67 ^av^
	a*	4.09 ± 0.23 ^ax^	3.91 ± 0.87 ^ay^	3.89 ± 0.33 ^av^	3.71 ± 0.26 ^ay^	3.87 ± 0.47 ^av^
	b*	5.12 ± 0.19 ^ax^	5.52 ± 0.33 ^ax^	5.52 ± 0.23 ^ax^	5.59 ± 0.33 ^ax^	5.38 ± 0.22 ^ax^

Letters a and b indicate means that differ significantly (*p* < 0.05) between different CAP treatment times within the same day. Letters v, w, x, y, and z indicate means that differ significantly (*p* < 0.05) through the different days of storage within the same treatment. Error bars indicate the standard deviation; each treatment time was repeated in triplicates, and each set was replicated two times, resulting in n = 6.

## Data Availability

The original contributions presented in the study are included in the article, further inquiries can be directed to the corresponding author.
